# Antibiotic Use in Febrile Children Presenting to the Emergency Department: A Systematic Review

**DOI:** 10.3389/fped.2018.00260

**Published:** 2018-10-08

**Authors:** Elles M. F. van de Voort, Santiago Mintegi, Alain Gervaix, Henriette A. Moll, Rianne Oostenbrink

**Affiliations:** ^1^Department of General Pediatrics, Erasmus MC-Sophia Children's Hospital, Rotterdam, Netherlands; ^2^Pediatric Emergency Department, Cruces University Hospital, University of the Basque Country, Bilbao, Spain; ^3^Division of Pediatric Emergency Medicine, Department of Child and Adolescent, Geneva University Hospitals and University of Geneva, Geneva, Switzerland

**Keywords:** pediatric emergency care, fever, children, antibiotic prescription, management

## Abstract

**Introduction:** While fever is the main complaint among pediatric emergency services and high antibiotic prescription are observed, only a few studies have been published addressing this subject. Therefore this systematic review aims to summarize antibiotic prescriptions in febrile children at the ED and assess its determinants.

**Methods:** We extracted studies published from 2000 to 2017 on antibiotic use in febrile children at the ED from different databases. Author, year, and country of publishing, study design, inclusion criteria, primary outcome, age, and number of children included in the study was extracted. To compare the risk-of-bias all articles were assessed using the MINORS criteria. For the final quality assessment we additionally used the sample size and the primary outcome.

**Results:** We included 26 studies reporting on antibiotic prescription and 28 intervention studies on the effect on antibiotic prescription. In all 54 studies antibiotic prescriptions in the ED varied from 15 to 90.5%, pending on study populations and diagnosis. Respiratory tract infections were mostly studied. Pediatric emergency physicians prescribed significantly less antibiotics then general emergency physicians. Most frequent reported interventions to reduce antibiotics are delayed antibiotic prescription in acute otitis media, viral testing and guidelines.

**Conclusion:** Evidence on antibiotic prescriptions in children with fever presenting to the ED remains inconclusive. Delayed antibiotic prescription in acute otitis media and guidelines for fever and respiratory infections can effectively reduce antibiotic prescription in the ED. The large heterogeneity of type of studies and included populations limits strict conclusions, such a gap in knowledge on the determining factors that influence antibiotic prescription in febrile children presenting to the ED remains.

## Introduction

Fever is the main complaint among pediatric emergency services (1). In only 15% (IQR 8·0–23·2%) a serious bacterial infection (SBI) is diagnosed with pneumonia and urinary tract infection (UTI) being the most prevalent (2, 3).

In contrast to the above, high antibiotic prescriptions are observed in febrile children (4, 5). Guidelines, or new diagnostic approaches have shown to effectively reduce antibiotic prescriptions in primary care (6–9). This is important because unnecessary antibiotic use increases antibiotic resistance (10, 11). In contrast to hospital based studies or primary care settings (11–15), few studies have been published in emergency department (ED) settings nor do we have valid estimates of potential benefits of antibiotic reducing interventions. Therefore our primary study aim is to assess antibiotic prescriptions for febrile children visiting the emergency department and their determinants. Secondary, we aim to investigate potential interventions that have been proven to be effective in the ED.

## Methods

### Study characteristics

All descriptive and interventional studies published in 2000–2017 reporting on antibiotic use in children (age under 18) with fever in the emergency department were eligible for this review.

### Search strategy

We searched Embase, Medline (OvidSP), Web-of-science, Scopus, Cinahl, Cochrane, PubMed publisher, and Google scholar for the (analogs of) keywords: fever, antibiotics, emergency department, children and antibiotic prescription. Initially search was performed in 2015 and updated in October 2017 (Supplementary Material 1). References were checked for additional articles to be included.

### Inclusion

A screening by title/abstract resulted in potential eligible articles that underwent full text review. Two authors reviewed all articles; any discrepancies were solved by oral agreement between authors.

– Setting: Emergency department; if mixed settings, at least 30% (50 patients minimum) of the population needed to be admitted to the ED.
– Design: observational studies and randomized controlled trials with a minimum of 50 participants.
– Outcome: the studies had to report the number or percentage of antibiotics prescribed.
– Population: participants under the age of 18; if mixed ages, at least 20% of the population needed to be <18 years (with a minimum of 50) or age specific antibiotic prescriptions had to be presented. Studies on children with specific comorbidities only were excluded.
– Fever: at least 30% of all included children needed to have fever or the reason of visit was (reported) fever.

### Quality assessment of included articles

To compare the risk-of-bias of all these different study designs all articles were assessed using the MINORS criteria (16). Zero points were given for the item if not reported, one point if reported but insufficient and two points if reported and sufficient. As loss to follow-up was not applicable, due to emergency setting, we have let this particular item out of consideration; the maximum score for studies is 14 or 22 for respectively non-comparative and comparative studies. A maximum score on the MINORS criteria was needed to receive the status of a low risk of bias study (A) (17). For the final quality assessment we additionally used the sample size and the primary outcome. A high quality study was defined by status low risk of bias (A) on the MINORS, antibiotic prescription being the primary outcome and a sample size of at least 500 children. Two reviewers (EV and RO) have independently assessed all included studies. Supplementary Material 2 contains the complete quality assessment.

### Data extraction and analysis

Extracted data included: Author, year, and country of publishing, study design, inclusion criteria, primary outcome, median (or mean when median not available) age, number of included children. Aiming to invest determinants of antibiotic prescription, we additionally extracted (if available): diagnosis, type of antibiotics, type of physicians, and type of intervention.

Due to heterogeneity in participants, outcome measures, interventions and study designs, no statistical pooling but a qualitative analysis was performed (18). Results are presented for the 5 main diagnosis, i.e., fever, AOM, pneumonia, other respiratory tract infections (RTI other) and UTI, with a minimum of 50 cases per diagnostic group required.

## Results

### Literature search

**Figure 1:**
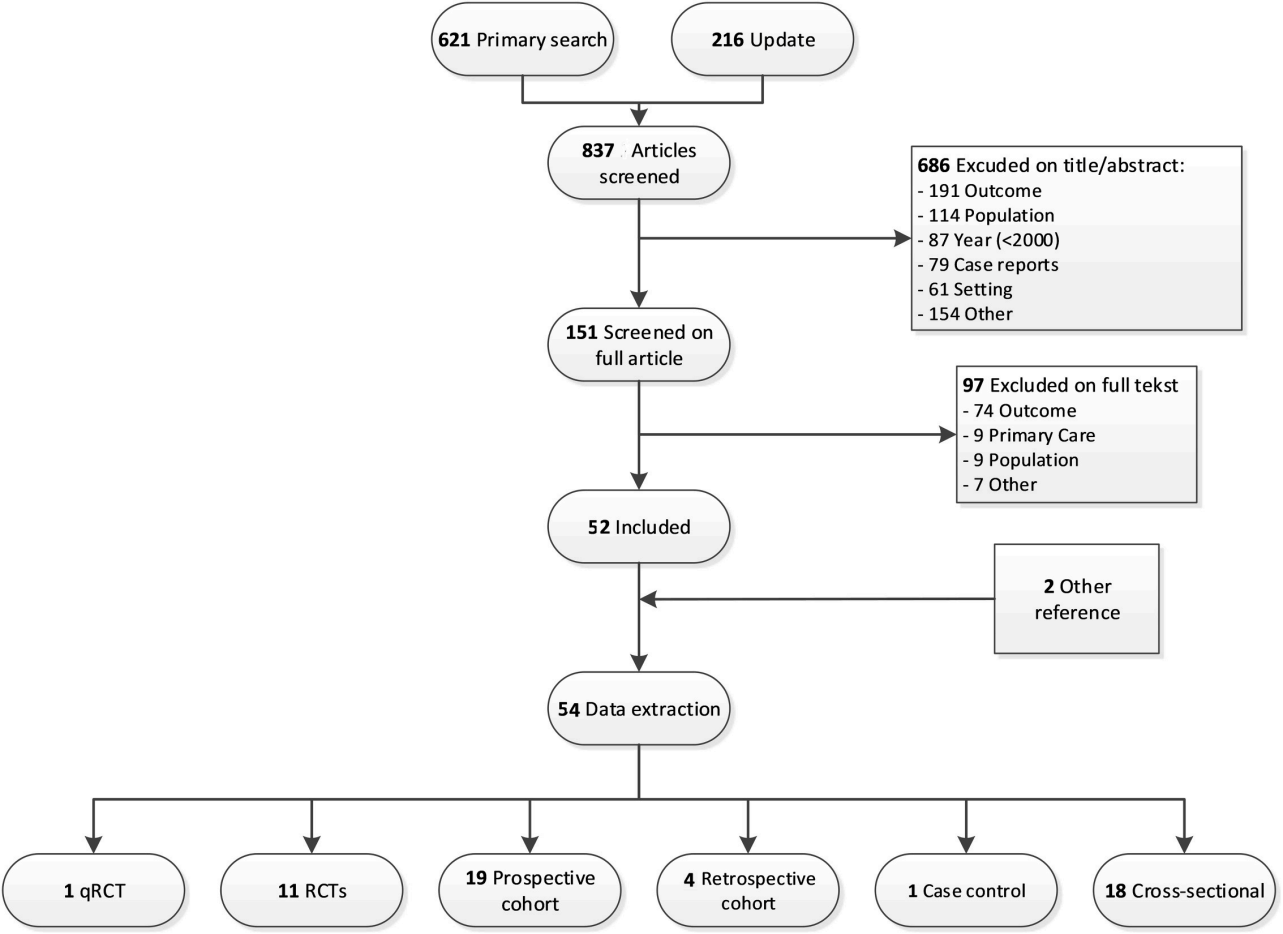
Flowchart of the study selection and exclusion.

We obtained 837 articles by literature search. Screening the full text articles excluded 97 out of 151, which leaves 52 articles for data extraction. Two additional studies were included by reference check of included studies (Figure 1).

### Characteristics of the included studies

The study characteristics are presented in Table 1 for the included 54 studies. Most studies come from the US (*n* = 32, 59%), 16 others came from Europe, and 6 others from Canada (*n* = 3) (33, 36, 49), Australia (*n* = 2) (3), and Israel (*n* = 1) (26). The size of the studied population varied between 72 and 266.000 participants (median = 391). Most studies included children up to 36 months (*n* = 14, 25%) or all ages < 18 year (*n* = 18, 32%). Antibiotic prescription was the primary outcome in 33 studies (59%). Quality and feasibility assessment of the included studies (Supplementary Material 2).

**Table 1:** Characteristics of descriptive studies about antibiotic prescription.

**Reference, Country**	**Study design**	**Age group/inclusion**	**Median (IQR) or Mean age ± SD**	**Inclusion criteria**	**N children included**	**Quality**
Ahmed et al. (19), US	CSp	0–18 years	NR	URTI	321	Low
Angoulvant et al. (20), France	CR	<18 years	17 months (7–40)	ARTI	53.055	High
Aronson et al. (21), US	CSr	29–56 days	46 days (37–53)	Fever	1626	High
			45 days (37–53)			
Ayanruoh et al. (22), US	CSr	3–18 years	NR	Clinical diagnosis of pharyngitis	8280	Low
Benin et al. (23), US	CSr	3–18 years	8.7 years (6–13)	Diagnosis pharyngitis	391	Moderate
Benito-Fernández et al. (24), Spain	CP	0–36 months	6.86 months ± 6.3°	Fever without source	206	Low
			6.55 months ± 6.8°			
Blaschke et al. (25) US^◇^	CSr	All ages	53% < 18 years	Influenza	58	Low
Brauner et al. (26), Israel	CCr	3–36 months	NR	Fever and complete blood count	292	Moderate
Bonner et al. (27), US	RCT	2 months−21 years	NR	Influenza	202	Moderate
Bustinduy et al. (28), UK	CP	<16 years	2 years (1–4 years)	Fever or reported fever	1097	Moderate
Chao et al. (29), US	RCT	2–12 years	5.01 years (3.67–6.68)	AOM	206	Moderate
			3.73 years (2.82–5.75)			
Craig et al. (3), Australia	CP	<6 years	± 60% < 24 months	Fever	15.781	High
Coco et al. (30), US	CSr	<12 years	± 2 years^*^	AOM	8325	High
Colvin et al. (31), US	CP	2–36 months	8.0 months	Fever without source ¥	75	Low
Copp et al. (32), US	CSr	<18 years	±6 years^*^	UTI	1828 (36% in ED)	Low
Doan et al. (33), Canada	RCT	3–36 months	15 months (3–36)	Acue respiratory symptoms	199	Moderate
			14 months (4–34)			
Fischer et al. (34), US	CP	2–18 years	68% 2–6 years	AOM	144	Low
Galetto Lacour et al. (35), Switzerland	CP	7 days −36 months	11 months^*^	Fever without source ¥	124	Moderate
Galetto-Lacour et al. (35), Switzerland	CP	7 days −36 months	7.2 months (0.4–31.1)	Fever without source ¥	99	Low
			9.7 months (0.7–34)			
Goldman et al. (36), Canada	CP	<3 months	48.7 days ± 23.6°	Fever	257	Low
Houten et al. (37), Netherlands	CP	2–60 months	21 months ± 16°	Fever and LRTI symptoms or without source	577	Moderate
Irwin et al. (38), UK	CP	<16 years	2.4 years (0.9–5.7)	Fever and blood tests	1101	High
Isaacman et al. (39), US	CR	3–36 months	18 months ± 9.8°	Fever without source in a GED¥	79	Low
			16.3 months ±8.8°	Fever without source in a PED¥	498	
Iyer et al. (40), US	RCT	2–24 months	±75% 6–24 months	Fever	700	Moderate
Jain et al. (41), US	CP	<18 years	NR	Fever	19075	High
Khine et al. (42), US	CR	3–36 months	15.2 months ±8.7°	Reported fever in GED	237	Moderate
						
		3–36 months	16.6 months ±9.1°	Reported fever in PED	224	
Kilic et al. (43) Turkey	CSr	3–140 months	41.2 months ±31°	Asthma, croup, Bronchiolitis	2544	Low
Kornblith et al. (44), US	CSr	0–18 years	± 56% 1–5 years	ARTI	6461	High
Kronman et al. (45), US	CSr	1–18 years	50–60% 1–5 years	CAP	266.000	High
Lacroix et al. (46), France	RCT	7 days−36 months	3.4 months (1.5–10.4)	Fever without source	271	High
			4.8 months (1.7–10.4)			
Linder et al. (47), US	CSr	3–17 years	45% 6–11 years	Sore throat	6955	High
Li-Kim-Moy et al. (48), Australia	CR	0 ≤ 18 years	3.1 years (1.1–7.4)	Lab proven influenza	301	Moderate
Manzano et al. (49), Canada	RCT	1–36 months	12 ± 8 months°	Fever	384	High
			12 ± 8 months°			
Massin et al. (50) Belgium	CP	1–36 months	13.8 months ±9.7°	Fever without source ¥	376	Moderate
						
McCaig et al. (51), US	CSr	3 months−2 years	NR	Fever and BC (discharged)	5.4% of all ED visits	Low
McCormick et al. (52), US	RCT	6–72 months	±60% < 1 years	AOM	209	Moderate
Murray et al. (53), US	CP	<56 days	36 days ± 13.8	Fever	520	Low
Nelson et al. (54), US^*^	CP	3 months−18 years	2.8 years (4.4)	Pneumonia	3220	High
Nibhanipudi et al. (55), US^*^	CP	2–17 years	5.72 years ± 0.38° (m)	AOM	100	Low
			7.41 years ± 0.75° (f)			
Ochoa et al. (56), Spain	CSr	0–18 years	±3 years (1 months−18 years)	ARTI	6249	High
Ong et al. (57), US	CP	All ages (20% child)	33 years	URTI	272	Moderate
Özkaya et al. (58), Turkey	CSp	3–14 years	5.7 years ± 3.4°	Influenza like illness	97	Low
			4.25 years ± 2.02			
Ouldali et al. (59), France	qRCT	<18 years	1.6 years (0.7–3.6)	ARTI	196.062	High
			1.7 years (0.7–3.7)			
Planas et al. (60), Spain	CP	<3 months	35 days ± 31°	Fever without source and BC (admitted) ¥	381	Moderate
Ploin et al. (61), France	CP	<36 months	NR	Fever during influenza season	538	Moderate
Poehling et al. (62), US	RCT	<5 years	NR	Fever or ARS during influenza season	305	Moderate
Shah et al. (63), US	CSr	1–18 years	± 63% 1–4 years	Fever and cough or respiratory distress	3466	Moderate
Sharma et al. (64), US	CSr	2–24 months	9 months °	Fever and positive influenza test	72	Low
Spiro et al. (65), US	RCT	6–35 months	17.3 months°	Fever or ARS	681	High
			17.2 months°			
Spiro et al. (66), US	RCT	6 months−12 years	3.2 years	AOM	283	High
			3.6 years			
Trautner et al. (67), US	CSp	<18 years	17 months (11–25 months)	Hyperpyrexia	103	Moderate
de Vos-Kerkhof et al. (68), Netherlands	RCT	1 months−16 years	1.7 years (0.8–3.9)	Fever	439	Moderate
			2.0 years (1.0–4.2)			
Waddle and Jhaveri, (69), US	CSr	3–36 months	17 months ± 11°	FWS and BC	423	Low
			15 months ± 10°			
Wheeler et al. (70), US	CP	≤ 18 years	3 years (1 months−20 years)	Viral infections	144	Moderate

*CC, case control; CP, prospective cohort; CR, retrospective cohort; CS, cross-sectional; r, retrospective; p, prospective*.

* *Estimated/calculated from numbers in article*.

° *Mean age is given, median age was not reported*.

¥ *Fever without source: as defined in corresponding study*.

Sixteen studies (29%) were considered as high quality and 17 (30%) were considered low quality. In general, observational studies did not describe sufficiently how sample size was approximated. Almost all high quality studies, except one (3), used antibiotic prescriptions as a primary outcome.

### Antibiotic prescriptions in febrile children and specific conditions

Table 2 presents the antibiotic prescriptions among the five diagnostic groups we distinguished. Sixteen out of 26 descriptive studies focused on febrile children in general, one paper specifically addressed acute otitis media (AOM) (30), two pneumonia (45, 63), four other respiratory infections (RTI other)(19, 23, 43, 57), and one urinary tract infections (UTI)(32). One paper on febrile children also provide separate numbers for pneumonia and UTI (3) and one for AOM (61). Two additional papers focused on respiratory infections and provided separate numbers for pneumonia, AOM and RTI other (44, 56).

**Table 2:** Antibiotic prescription per diagnosis.

**Reference, Country**	**Age group/ inclusion**	**Median (IQR) or Mean age ± SD**	**Inclusion criteria**	**N children included**	**N antibiotics, % of study population^ł^**
**FEVER IN GENERAL**					
Bustinduy et al. (28), UK	<16 years	2 years (1–4 years)	Fever or reported fever	1097	44%
Colvin et al. (31), US	2–36 months	8.0 months	Fever without source ¥	75	45%
Craig et al. (3), Australia	<6 years	± 60% < 24 months	Fever	15.781	27%
Galetto Lacour et al. (35), Switzerland	7 days−36 months	11 months^*^	Fever without source ¥	124	62.1%
Galetto-Lacour et al. (35), Switzerland	7 days−36 months	7.2 months (0.4–31.1) 9.7 months (0.7–34)	Fever without source ¥	99	71%
Goldman et al. (36), Canada	<3 months	48.7 days ± 23.6°	Fever	257	55%
Houten et al. (60), Netherlands	2–60 months	21 months ± 16°	Fever and LRTI symptoms or without source	577	39%
Isaacman et al. (39), US	3–36 months	18 months ± 9.8°	Fever without source in a GED¥	79	39.2%
		16.3 months ±8.8°	Fever without source in a PED¥	498	16.7%
Khine et al. (42), US	3–36 months	15.2 months ±8.7°	Reported fever in GED	237	41%
	3–36 months	16.6 months ±9.1°	Reported fever in PED	224	27%
Massin et al. (50), Belgium	1–36 months	13.8 months ± 9.7°	Fever without source ¥	376	15%
Ploin et al. (61), France	<36 months	NR	Fever during influenza season	538	34.8%
**FEVER AND SELECTION ON ADDITIONAL TESTING OR CHARACTERISTICS**					
Irwin et al. (38), UK	<16 years	2.4 years (0.9–5.7)	Fever and blood tests	1101	855, 78%
Trautner et al. (67), US	<18 years	17 months (11–25 months)	Hyperpyrexia	103	46, 61.3%
Brauner et al. (26), Israel	3–36 months	NR	Fever and complete blood count	292	148, 50.7%
Planas et al. (60), Spain	<3 months	35 days ± 31°	Fever without source and BC (admitted) ¥	381	281, 73.8^*^%
**AOM**					
Coco et al. (30), US	<12 years	± 2 years^*^	AOM	8325	82.6%
Kornblith et al. (44), US	0–18 years	± 56% 1–5 years	AOM	647	88%
Ochoa et al. (56), Spain	0–18 years	±3 years (1 months−18 years)	AOM	821	93%
Ploin et al. (61), France	<36 months	NR	Fever during influenza season	18	89%
**PNEUMONIA**					
Craig et al. (3) Australia	<6 years	± 60% < 24 months	Pneumonia	533	69%
Kornblith et al. (44), US	0–18 years	± 56% 1–5 years	Pneumonia	657	86%
Kronman et al. (45), US	1–18 years	50–60% 1–5 years	CAP	266.000	86.1%
Ochoa et al. (56), Spain	0–18 years	±3 years (1 months−18 years)	Pneumonia	288	93%
Shah et al. (63), US	1–18 years	± 63% 1–4 years	Pneumonia	347	82%
**RTI OTHER**					
Ahmed et al. (19), US	0–18 years	NR	URTI	321	43%
Benin et al. (23), US	3–18 years	8.7 years (6–13)	Diagnosis pharyngitis	391	23%
Kilic et al. (43), Turkey	3–140 months	41.2 months ±31°	Asthma, croup, Bronchiolitis	2544	16.6%
Kornblith et al. (44), US	0–18 years	± 56% 1–5 years	URTI	5157	36%
Ochoa et al. (56), Spain	0–18 years	±3 years (1 months−18 years)	URTI	5140	51%
Ong et al. (57), US	All ages (20% child)	33 years	URTI	272	83, 31%
**UTI**					
Copp et al. (32), US	<18 years	±6 years^*^	UTI	1828	70%
Craig et al. (3), Australia	<6 years	± 60% < 24 months	Fever	543	66%

* *Estimated/calculated from numbers in article*.

° *Mean age is given, median age was not reported*.

¥ *Fever without source: as defined in corresponding study*.

ł *Antibiotic prescription is given for reported age group, except for Ong et al (57) antibiotic use for all ages is given*.

#### Fever

Sixteen out of 26 studies focused on febrile children in general, seven of them selected children based on fever without source; five included febrile children based on additional testing (Table 2). In studies of general febrile populations only, antibiotic prescriptions ranged from 15 to 71% (3, 31, 35, 36, 39, 42, 50, 61, 71). The lowest prescriptions (15%) came from a study on parenteral empirical antibiotics only (50). Study quality did not influence antibiotic prescription rate.

**Figure 2:**
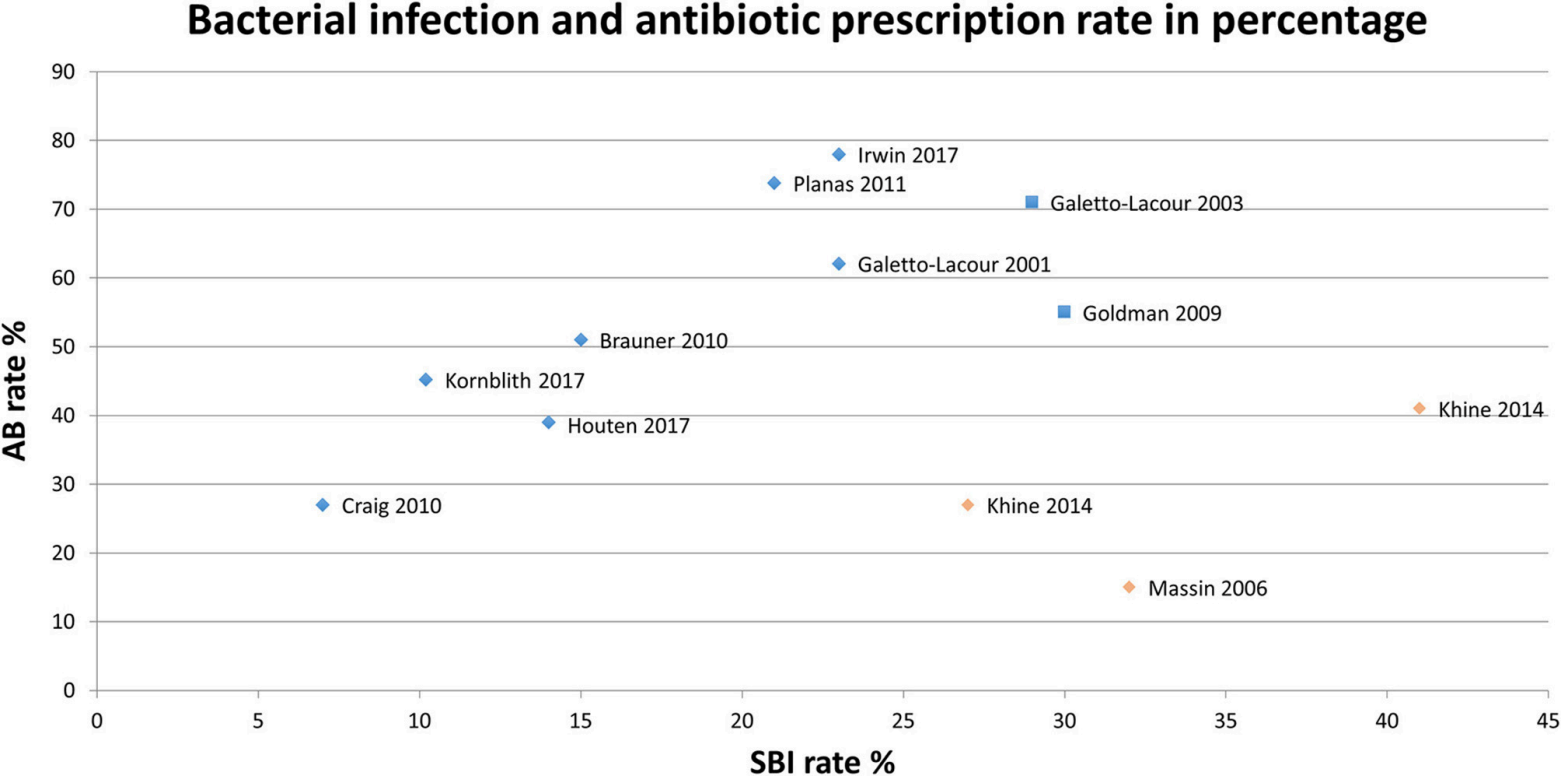
Serious bacterial infection rate and antibiotic prescriptions per study. 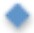 *High/Moderate quality*, 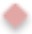 *High/Moderate quality, outlier*, 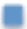 *Low quality*, 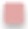 *Low quality, outlie*.

Three high quality, six moderate quality and two low quality studies reported on SBI rate, which ranged from 7 to 41% (Figure 2) (3, 26, 35–38, 42, 44, 50, 60, 71). As the SBI rate in Khine et al. (42) is similar to antibiotic prescriptions, one may question how SBI is defined. Massin et al. (50) reports on parenteral antibiotics only and may not represent antibiotic prescription in total. Focusing on the remaining eight studies, we observe a trend toward higher antibiotic prescriptions with higher rates of SBI, although not significant.

**Figure 3:**
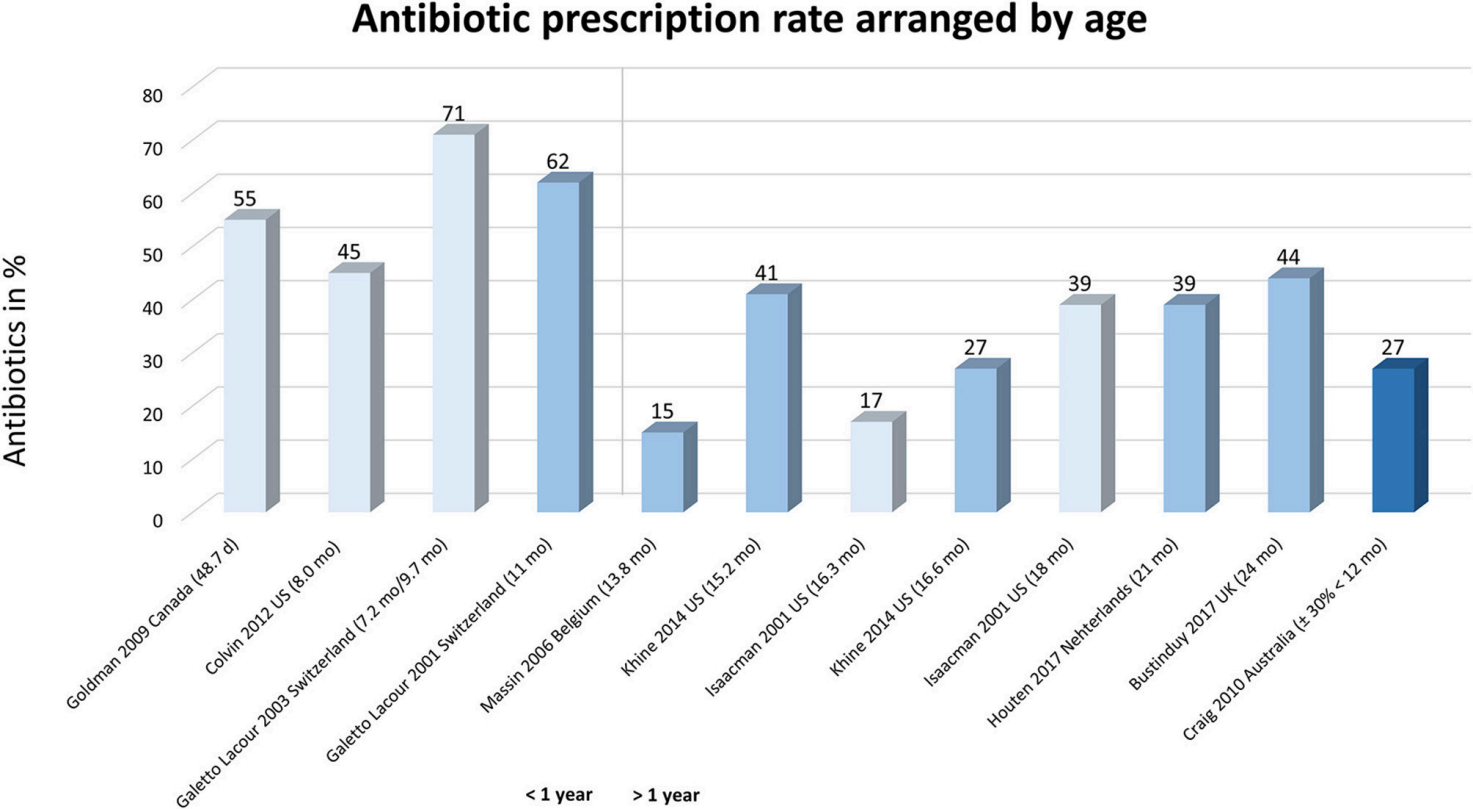
Antibiotic prescriptions arranged on age in children with fever. Studies are arranged by age, i.e., left represents younger children to right (older ages). Light bars represent studies with a low quality.

In the studies on fever in general, we observed a higher prescriptions in children under the age of one (45 to 71%; weighted mean 58%), compared to older ones (prescriptions of 17 to 44%; weighted mean 28%), independent of study quality (Figure 3) (3, 28, 31, 35–37, 39, 42, 50, 71).

None of the studies on febrile children in general compared antibiotic prescriptions between countries. In the eleven studies (3, 28, 31, 35–37, 39, 42, 50, 61, 71) on children with fever in general (without additional testing), the highest prescriptions were reported in a Swiss study (71%) (35) and the lowest in a study originating from the US (17%) (39). The three studies originating from the US reported antibiotic prescription between 39–45% (31, 39, 42); for the two Swiss studies this varied from 62 to 71%, although originating from the same hospital (35, 71).

#### Antibiotic prescription for specific diagnoses

Four studies provided data for antibiotic prescription in AOM, ranging from 88–93%. We could not determine influences of age on prescriptions. Five studies reported on antibiotic prescription in pneumonia, ranging from 69 to 93%. The study with the lowest prescription (3) included children <6 years only compared to the other four (including children in the range of 1-18 years). Antibiotic prescription in RTI other (6 studies) varied on a broader range from 17 to 51%, but could not be related to age. Only two studies provided information on antibiotic prescription in UTI, ranging from 66 to 70%.

#### Type of antibiotic prescription

**Figure 4:**
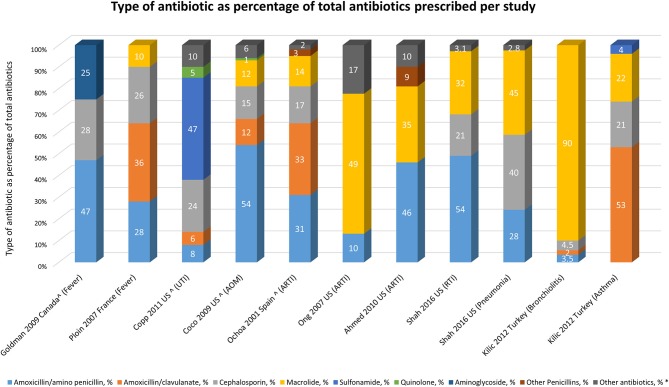
Type of antibiotic as percentage of total antibiotics prescribed per study. *As defined in article. Ahmed et al. (19): not specified; Copp et al. (32): nitrofurantoin and others are not specified antibiotics. Coco et al. (30): not specified. Ochoa et al. (56): trimethoprim/sulfamethoxazole, clindamycin, fosfomycin, rifampin, trimethoprim, topical use and others are not specified. Ong et al. (57): trimethoprim/sulfamethoxazole; Shah et al. (63): not specified. ^∧^Calculated from article as percentage of total antibiotics, in article given as percentage of cases.

Nine out of 26 (35%) studies [two high quality (30, 56)] reported on antibiotic type (Figure 4). Six studies addressed respiratory tract infections (19, 30, 43, 56, 57, 63) and five were conducted in the US (19, 30, 32, 57, 63). We did not observe a predominance for one antibiotic type for a specific diagnosis or country; amoxicillin was always reported. Studies describing cephalosporin use (*n* = 7) included both second or third generations.

#### Prescribing physician

Five (39, 42, 47, 63, 72) out of seven studies [three high quality studies (44, 47, 66)], reported significant lower antibiotic prescriptions by pediatric emergency physicians compared to general emergency physicians (Table 3). Two addressed young children with fever without source (39, 42), and five addressed older children with respiratory tract infections (19, 44, 47, 63, 65).

**Table 3:** Difference in antibiotic prescription between general physicians and pediatric physicians.

**Reference, Country**	**N Antibiotics given by GEMP/N seen by GEMP % antibiotics**	**N antibiotics given by PEMP/N seen by PEMP % antibiotics**	**Inclusion criteria**
Isaacman et al. (39), US	37/79, 39%	83/498, 17%	FWS
Khine et al. (42), US	97/237, 41%	61/224, 27%	FWS
Ahmed et al. (19), US	NR/238, 32%	NR/345, 17%	URTI
Kornblith et al. (44), US	NR, 46%	NR, 42%	ARTI
Shah et al. (63), US	2946, 50%	520, 35%	Febrile RTI
Linder et al. (47), US	NR, 60%	NR, 47%	Sore throat
Spiro et al. (65), US^*^	NR, 30%	NR, 26%	Fever/ARS

* *No significant statistical difference was found*.

High quality study.

Moderate quality study.

Low quality study.

### The effect of interventions on antibiotic prescription

Nine out of 27 studies on interventions for antibiotic prescription (32%) reported about rapid viral testing (22, 24, 25, 27, 33, 40, 58, 62, 64), four about delayed antibiotic prescription in acute otitis media (29, 34, 52, 66), six about guideline/management strategies (20, 21, 41, 53, 59, 68), four about laboratory tests (22, 46, 47, 49) and five using other interventions (Table 4). In fourteen studies (50%) a significant reduction in antibiotic use was found.

**Table 4:** Influence of intervention on antibiotic prescription.

**Reference, Country**	**Median (IQR) or Mean age ± SD ¥**	**Intervention**	**Inclusion**	**N intervention total, % AB**	**N controls total, % AB**
**FEVER IN GENERAL**					
Aronson et al. (21), US	46 days (37–53)	CPG recommending ceftriaxone compared to no CPG	Fever	306, 64.1%^∧^	1.304, 11.7%^∧^
	45 days (37–53)				
		CPG recommending against ceftriaxone compared to no CPG		313, 10.9%^∧^	1.304, 11.7%^∧^
Jain et al. (41), US	NR	Physician feedback through scorecards	Fever	8.961, 10.8%	1.0114, 12%
Lacroix et al. (46), France	3.4 months (1.5–10.4)	Lab Score	FWS	131, 41.2%	140, 42.1%
	4.8 months (1.7–10.4)				
Manzano et al. (49), Canada	12 ± 8 months°	PCT testing	Fever	192, 25%	192, 28%
	12 ± 8 months°				
Murray et al. (53), US	36 days ± 13.8	Implementation of a clinical pathway	Fever	296, 69%	224, 72%
de Vos-Kerkhof et al. (68), Netherlands	1.7 years (0.8–3.9)	Clinical decision model	Fever	219, 35.6%	220, 41.8%
	2.0 years (1.0–4.2)				
**(SUSPICION OF) BACTERIAL INFECTIONS**					
Nelson et al. (54), US ^*^	2.8 years (4.4)	Antibiotic prescription rate before and after CXR result	Pneumonia	1610, 23%	1610, 7%
de Vos-Kerkhof et al. (68), Netherlands	1.8 (0.9–4.1)	Clinical decision model	Fever and SBI	192, 22.9%	192, 27.1%
Waddle and Jhaveri (69), US	17 months ± 11°	PCV7	FWS and BC	275, 57.2%	148, 60.8%
	15 months ± 10°				
**INFLUENZA**					
Blaschke 2014 (25), US^◇^	53% < 18 years	Rapid viral testing (positive/negative RVT)	RVT performed	NR, 11%	NR, 47%
Benito-Fernández et al. (24), Spain	6.86 months ± 6.3°	Rapid viral testing (positive/negative RVT)	Fever without source	84, 0%	122, 38.5%
	6.55 months ± 6.8°				
Bonner et al. (27), US	NR	Rapid viral testing (RVT /no RVT)	Influenza positive	96, 7%	106, 25%
Doan et al. (33), Canada	15 months (3–36)	Rapid viral testing (POCT/standard testing)	Acute respiratory symptoms	89, 18%	110, 21%
	14 months (4–34)				
Iyer et al. (40), US	±75% 6–24 months	Rapid viral testing (RVT/ no RVT)	Fever	345, 25.3%	355, 30.5%
Li-Kim-Moy et al. (48), Australia	3.1 years (1.1–7.4)	Rapid viral testing (POCT/standard testing)	Lab proven influenza	236, 33%	65, 54%
Özkaya et al. (58), Turkey	5.7 years ± 3.4°	Rapid viral testing (RVT /no RVT)	Influenza-like illness	50, 58%	47, 100%
	4.25 years ± 2.02°				
Poehling et al. (62), US	NR	Rapid viral testing (RVT/no RVT)	Fever or ARS during influenza season	135, 32%	170, 29%
Sharma et al. (64), US	9 months°	Rapid viral testing (RVT /no RVT)	Fever and positive influenza test	47, 2%	25, 24%
**AOM**					
Chao et al. (29), US	5.01 years (3.67–6.68)	Delayed prescription with and without prescription	AOM	100, 19%	106, 46%
	3.73 years (2.82–5.75)				
Fischer et al. (34), US	68% 2–6 years	Wait-and-see prescription in AOM	AOM	144, 27%	N.A.
McCormick et al. (52), US	±60% < 1 years	Wait-and-see prescription in AOM	AOM	100, 34%	109, 100%
Nibhanipudi et al. (55), US^*^	5.72 years ± 0.38° (m)	WBC >15.000 or WBC < 15.000	AOM	93, 3%	7, 100%
	7.41 years ± 0.75° (f)				
Spiro et al. (66), US	3.2 years	Wait-and-see prescription in AOM	AOM	138, 38%	145, 87%
	3.6 years				
**RTI Other**					
Angoulvant et al. (20), France	17 months (7–40)	Implementing guidelines	ARTI	NR, 21%	NR, 32.1%
Ayanruoh et al. (22), US	NR	Rapid streptococcal testing	Clinical diagnosis of pharyngitis	6.557, 22.45%	1.723, 41.38%
Linder et al. (47), US	45% 6–11 years	GABHS testing in sore throat	Sore throat	NR, 48%	NR, 51%
Ouldali et al. (59), France	1.6 years (0.7–3.6)	Implementation of national guidelines	ARTI	134.450,−28.4%	61.612
	1.7 years (0.7–3.7)				
Spiro et al. (65), US	17.3 months°	Tympanometry for reduction antibiotics in AOM	Fever or ARS	341, 28.8%	340, 26.8%
	17.2 months°				
Wheeler et al. (70), US	3 years (1 months−20 years)	Videotape in waiting room	Viral infections	71, 4.2%	73, 6.8%

∧ *Only parenteral antibiotic prescription rate is given. Highlighted studies indicate studies with significant results*.

* *Estimated/calculated from numbers in article*.

° *Mean age given, median age not reported*.

#### Interventions for AOM

Interventions with a significant effect on antibiotic reduction were guidelines and the wait-and-see prescription in acute otitis media (AOM). For this latter a significant reduction was found in four articles (three of them with moderate to high quality) (29, 34, 52, 66).

#### Viral testing intervention

Most studies on interventions for reduction of antibiotic prescription addressed rapid viral testing for influenza (RVT, *n* = 9). Fewer antibiotics were prescribed when the RVT is positive (24, 25, 27, 64), although not confirmed by studies on the impact of RVT use vs. not using RVT in the ED (27, 40, 58, 62). Only one low quality study reported a significant difference for this topic (58). The use of point-of-care testing above testing on indication had only significant benefit in children with proven influenza (33, 48). One study reported reduced length of stay, but no effect on antibiotic prescription (48).

#### Other interventions

Three high quality studies showed a significant reduction in antibiotic prescription by a guideline for lower respiratory infections or infants with fever (20, 21, 41). Among two articles on streptococcal A testing, the article with the highest quality didn't find a significant reduction (22, 47). Introduction of a clinical pathway for young febrile infants showed reduced time to first antibiotic dose, but did not evaluate the effect on antibiotic prescription itself (53). The use of chest radiographs in particular reduces antibiotics in children with low clinical suspicion of pneumonia (54). For all other interventions no significant reduction was found on antibiotic prescription (46, 49, 65, 69, 70).

## Discussion

### Interpretation of main findings

We observed a highly variable reported antibiotic prescriptions in children presenting to a general or pediatric ED in the five major groups of diagnosis. Studies on a specific diagnosis, such as AOM, pneumonia, or UTI report higher antibiotic prescriptions. However, studies are too heterogeneous to study true effects of determinants. Strong evidence was found for watchful waiting in AOM and implementation of guidelines for fever or respiratory infections to reduce antibiotic use in the ED. Intervention studies report mostly on rapid viral testing for influenzae (RVT) to reduce antibiotic prescription, but its effect is controversial.

It is important to note that the high variability in antibiotic prescription observed in our systematic review differ from reported antibiotic prescriptions from literature, or websites (12, 73). However, these numbers are based on national or local registries and include in-hospital patients, not reflecting our interest on use of antibiotics in ED settings. Next, not all countries are represented in our systematic review and only Switzerland, USA are represented by more than one study. For the latter two, however we observed high variability in antibiotic prescription within studies of the same country. Even within studies focusing on similar group of diagnoses, we observed a large heterogeneity in their way of patient selection and their type of febrile illness. Therefore, we think these antibiotic prescriptions cannot be considered to be representative for the general population of febrile children in a country.

Limited evidence was found for age effects on antibiotic prescriptions, potentially due to age distribution among study populations. Infants below 2 months are underrepresented in our review. From community studies, we know that pre-school children are more frequently exposed to antibiotic therapy (13).

After exclusion of two outlier studies given their patient selection and outcome definition (42, 50), we observed in studies on children with fever a trend toward higher antibiotic prescriptions in studies with higher SBI rates is noticeable. This, however, only explains some variation in antibiotic prescription.

Similar to studies in primary care, watchful waiting intervention seems highly effective for reducing antibiotic use in AOM at the ED (74). Results however are limited to patients above the age of 6 months that did not appear toxic and it is questionable if the study populations were large enough to detect serious adverse outcomes such as meningitis. Although the most frequently studied intervention, rapid viral testing for influenza has no additional effect above testing on indication and controversial evidence was found for its effect. Effects of guidelines are seen in two well-defined groups (respiratory infections or young febrile infants) and including a well-defined implementation plan. Implementation of a clinical decision model to reduce antibiotic prescriptions was only tested in a tertiary pediatric university ED and antibiotic reduction was not a primary outcome of this study (17). All other interventions are not (yet) proven to be effective for reducing the antibiotic prescriptions in children on the ED. Overall the evidence to reduce antibiotic prescription in the emergency department remains limited. We observed a general association between antibiotic prescription and the type of prescriber, i.e., pediatricians prescribe less antibiotics than general physicians may suggest that guideline implementation could be most effective in hospitals with general physicians treating children in the ED.

### Limitations

The quality of the studies that reported about fever in general was low to moderate, with only one high quality study (3). Specific drawbacks of study design are included in the MINOR assessment as a measure of quality. The use of MINORS in combination with the study population and study aim helps to increase the reproducibility of this review and made it possible to compare the different levels of evidence (16). Most studies did not reported on missing values regarding antibiotic prescription, which could lead to an underestimation of antibiotic prescriptions. In a substantial part of the included papers, antibiotic prescription was not the primary outcome. This may explain some diversity in antibiotic prescriptions, although this was partially corrected for in the quality assessment.

This systematic review focuses on prescription of antibiotics in the ED setting. In many European countries, antibiotics are available as over the counter drugs as well (75). This issue is not accounted for by any of the articles, which may lead to a general underestimation of the antibiotic use.

Unfortunately, we observed a large heterogeneity of the studies or had only 1 study per diagnosis group, hampering meta-analysis. Most heterogeneity is caused by specific patient selection (age, setting), by study design (intervention vs. observational cohort study). This also applies to the population of febrile children <36 months that constitute the majority of ED attendances.

### Future research recommendations

To validly estimate baseline antibiotic prescriptions in children with fever presenting to the emergency department we need observational studies including the general spectrum of febrile children. Being able to determine influences of antibiotic prescription, we should address geographical and cultural influences, differences in setting, adherence area, general patient characteristics, and descriptors of illness severity. Insight in these determinants may help to define targets for intervention to reduce antibiotic prescriptions. Next, this information will contribute to valid power calculations for intervention studies and to generalize effects to other settings.

## Conclusion

A summary of studies on antibiotic prescription in the 5 main diagnostic groups at the ED did not yield uniform outcomes. There seems to be a trend toward higher antibiotic prescriptions in younger children and for diagnoses that are more often related to bacterial infections. Delayed antibiotic prescription in children with acute otitis media and guidelines for fever/LRTI seem useful to reduce antibiotic prescriptions at the ED. However no strict conclusions can be drawn on the basis of this review because of the large heterogeneity of type of studies and included populations. This means that there is still a gap in knowledge on the determining factors that influence antibiotic prescription in febrile children presenting to the ED. A multicentre study including a wide range of countries on a general population of febrile children would be recommended to provide a valid baseline of antibiotic prescriptions in general, and influencing factors that identify targets for future interventions.

## Author contributions

EvdV was responsible for search, dataextraction and writing of the manuscript. HM, SM, and AG contributed to datainterpretation and writing of the manuscript. RO concepted the idea of the paper, supervised search, dataextraction, and writing of the manuscript.

### Conflict of interest statement

The authors declare that the research was conducted in the absence of any commercial or financial relationships that could be construed as a potential conflict of interest.

## Abbreviations

AB: antibiotic(s)
AOM: acute otitis media
ARS: acute respiratory symptoms
ARTI: acute respiratory tract infection
BC: blood culture
CAP: community acquired pneumonia
CC: case control study
CI: confidence interval
CP: cohort study prospective
CR: cohort study retrospective
CS: cross sectional study
CSF: cerebrospinal fluid
d: days
ED: emergency department
EL: extreme leukocytosis
FWS: fever without source
GED: general emergency department
GEMP: general emergency medicine physician
ILI: influenza-like illness
ML: moderate leukocytosis
mo: months
NR: not reported
NS: not specified
PED: pediatric emergency department
PEMP: pediatric emergency medicine physician
qRCT: quasi-randomized controlled trial
RCT: randomized controlled trial
reg: registration
RIDT: rapid influenza diagnostic tests
RST: rapid streptococcal test
RVT: rapid viral testing
SBI: serious bacterial infection
SD: standard deviation
T: temperature
URTI: upper respiratory tract infection
UTI: urinary tract infection
y: years.
