# Supplementary material for: Antibiotic Use in Febrile Children Presenting to the Emergency Department: A Systematic Review
Source: Front Pediatr. 2018 Oct 8;6:260. doi: 10.3389/fped.2018.00260 (PMC6186802; doi:10.3389/fped.2018.00260)
Supplement: Supplementary file 1 [file Data_Sheet_1.docx]

# Supplementary material

Supplementary material 1. Search strategy

**Embase.com**

('antibiotic agent'/exp OR 'antiinfective agent'/de OR 'antibiotic therapy'/exp OR (antibiotic* OR antiinfect* OR (anti NEXT/1 (biotic* OR infect*)) OR ceftriaxone* OR amoxicillin* OR penicillin* OR vancomycin* OR cotrimoxazole* OR ampicillin* OR gentamicin* OR clindamycin* OR erythromycin* OR cefotaxime* OR ciprofloxacin* OR dexamethasone* OR prednisone* OR azithromycin* OR cytarabine* OR cyclophosphamide* OR macrolide* OR doxycycline*):ab,ti) AND (fever/exp OR hyperthermia/exp OR (fever OR febril* OR hypertherm*):ab,ti) AND ('emergency ward'/exp OR 'emergency treatment'/de OR 'emergency care'/de OR 'emergency nursing'/de OR 'evidence based emergency medicine'/de OR 'emergency health service'/exp OR 'emergency medicine'/exp OR 'emergency physician'/exp OR (emergenc* OR (acute near/3 (care OR medicine))):ab,ti) AND (child/exp OR newborn/exp OR adolescent/exp OR adolescence/exp OR pediatrics/exp OR childhood/exp OR 'child health'/de OR 'child health care'/exp OR 'child care'/exp OR 'pediatric ward'/de OR 'pediatric hospital'/de OR (adolescen* OR infan* OR newborn* OR (new NEXT/1 born*) OR baby OR babies OR neonat* OR child* OR kid OR kids OR toddler* OR teen* OR boy* OR girl* OR minors OR underag* OR (under NEXT/1 ag*) OR juvenil* OR youth* OR kindergar* OR puber* OR peripuber* OR pubescen* OR prepubescen* OR prepubert* OR pediatric* OR paediatric* OR school* OR preschool* OR highschool*):ab,ti) AND ('drug use'/de OR prescription/de OR 'inappropriate prescribing'/de OR (((drug* OR antibiotic*) NEAR/3 (use OR usage OR registrat* OR consum* OR utiliz* OR pattern* OR reduc* OR decrease*)) OR prescri* OR overprescri* OR underprescri*):ab,ti)

**Medline (Ovid)**

(exp Anti-Bacterial Agents/ OR Anti-Infective Agents/ OR (antibiotic* OR antiinfect* OR (anti ADJ (biotic* OR infect*)) OR ceftriaxone* OR amoxicillin* OR penicillin* OR vancomycin* OR cotrimoxazole* OR ampicillin* OR gentamicin* OR clindamycin* OR erythromycin* OR cefotaxime* OR ciprofloxacin* OR dexamethasone* OR prednisone* OR azithromycin* OR cytarabine* OR cyclophosphamide* OR macrolide* OR doxycycline*).ab,ti.) AND (exp fever/ OR (fever OR febril* OR hypertherm*).ab,ti.) AND (exp Emergencies/ OR "Emergency Nursing"/ OR "emergency treatment"/ OR exp "Emergency Service, Hospital"/ OR exp "Emergency Medicine"/ OR exp "Emergency Medical Services"/ OR (emergenc* OR (acute ADJ3 (care OR medicine))).ab,ti.) AND (exp child/ OR exp infant/ OR adolescent/ OR exp pediatrics/ OR exp Child Health Services/ OR Hospitals, Pediatric/ OR (adolescen* OR infan* OR newborn* OR (new ADJ born*) OR baby OR babies OR neonat* OR child* OR kid OR kids OR toddler* OR teen* OR boy* OR girl* OR minors OR underag* OR (under ADJ (age* OR aging)) OR juvenil* OR youth* OR kindergar* OR puber* OR pubescen* OR prepubescen* OR prepubert* OR pediatric* OR paediatric* OR school* OR preschool* OR highschool*).ab,ti.) AND (exp Drug Utilization/ OR exp prescriptions/ OR Inappropriate Prescribing/ OR (((drug* OR antibiotic*) ADJ3 ("use" OR usage OR registrat* OR consum* OR utiliz* OR pattern* OR reduc* OR decrease*)) OR prescri* OR overprescri* OR underprescri*).ab,ti.)

**Cochrane**

((antibiotic* OR antiinfect* OR (anti NEXT/1 (biotic* OR infect*)) OR ceftriaxone* OR amoxicillin* OR penicillin* OR vancomycin* OR cotrimoxazole* OR ampicillin* OR gentamicin* OR clindamycin* OR erythromycin* OR cefotaxime* OR ciprofloxacin* OR dexamethasone* OR prednisone* OR azithromycin* OR cytarabine* OR cyclophosphamide* OR macrolide* OR doxycycline*):ab,ti) AND ((fever OR febril* OR hypertherm*):ab,ti) AND ((emergenc* OR (acute near/3 (care OR medicine))):ab,ti) AND ((adolescen* OR infan* OR newborn* OR (new NEXT/1 born*) OR baby OR babies OR neonat* OR child* OR kid OR kids OR toddler* OR teen* OR boy* OR girl* OR minors OR underag* OR (under NEXT/1 ag*) OR juvenil* OR youth* OR kindergar* OR puber* OR peripuber* OR pubescen* OR prepubescen* OR prepubert* OR pediatric* OR paediatric* OR school* OR preschool* OR highschool*):ab,ti) AND ((((drug* OR antibiotic*) NEAR/3 (use OR usage OR registrat* OR consum* OR utiliz* OR pattern* OR reduc* OR decrease*)) OR prescri* OR overprescri* OR underprescri*):ab,ti)

**Web-of-science**

TS=(((antibiotic* OR antiinfect* OR (anti NEAR/1 (biotic* OR infect*)) OR ceftriaxone* OR amoxicillin* OR penicillin* OR vancomycin* OR cotrimoxazole* OR ampicillin* OR gentamicin* OR clindamycin* OR erythromycin* OR cefotaxime* OR ciprofloxacin* OR dexamethasone* OR prednisone* OR azithromycin* OR cytarabine* OR cyclophosphamide* OR macrolide* OR doxycycline*)) AND ((fever OR febril* OR hypertherm*)) AND ((emergenc* OR (acute near/3 (care OR medicine)))) AND ((adolescen* OR infan* OR newborn* OR (new NEAR/1 born*) OR baby OR babies OR neonat* OR child* OR kid OR kids OR toddler* OR teen* OR boy* OR girl* OR minors OR underag* OR (under NEAR/1 age*) OR juvenil* OR youth* OR kindergar* OR puber* OR peripuber* OR pubescen* OR prepubescen* OR prepubert* OR pediatric* OR paediatric* OR school* OR preschool* OR highschool*)) AND ((((drug* OR antibiotic*) NEAR/3 (use OR usage OR registrat* OR consum* OR utiliz* OR pattern* OR reduc* OR decrease*)) OR prescri* OR overprescri* OR underprescri*)))

**Scopus**

TITLE-ABS-KEY(((antibiotic* OR antiinfect* OR (anti W/1 (biotic* OR infect*)) OR ceftriaxone* OR amoxicillin* OR penicillin* OR vancomycin* OR cotrimoxazole* OR ampicillin* OR gentamicin* OR clindamycin* OR erythromycin* OR cefotaxime* OR ciprofloxacin* OR dexamethasone* OR prednisone* OR azithromycin* OR cytarabine* OR cyclophosphamide* OR macrolide* OR doxycycline*)) AND ((fever OR febril* OR hypertherm*)) AND ((emergenc* OR (acute W/3 (care OR medicine)))) AND ((adolescen* OR infan* OR newborn* OR (new W/1 born*) OR baby OR babies OR neonat* OR child* OR kid OR kids OR toddler* OR teen* OR boy* OR girl* OR minors OR underag* OR (under W/1 age*) OR juvenil* OR youth* OR kindergar* OR puber* OR peripuber* OR pubescen* OR prepubescen* OR prepubert* OR pediatric* OR paediatric* OR school* OR preschool* OR highschool*)) AND ((((drug* OR antibiotic*) W/3 (use OR usage OR registrat* OR consum* OR utiliz* OR pattern* OR reduc* OR decrease*)) OR prescri* OR overprescri* OR underprescri*)))

**CINAHL**

(MH Antibiotics+ OR MH AntiInfective Agents+ OR (antibiotic* OR antiinfect* OR (anti N1 (biotic* OR infect*)) OR ceftriaxone* OR amoxicillin* OR penicillin* OR vancomycin* OR cotrimoxazole* OR ampicillin* OR gentamicin* OR clindamycin* OR erythromycin* OR cefotaxime* OR ciprofloxacin* OR dexamethasone* OR prednisone* OR azithromycin* OR cytarabine* OR cyclophosphamide* OR macrolide* OR doxycycline*)) AND (MH fever+ OR (fever OR febril* OR hypertherm*)) AND (MH Emergencies+ OR MH "Emergency Nursing+" OR MH "Emergency Treatment (Non-Cinahl)+" OR MH "Emergency Service+" OR MH "Emergency Medicine+" OR MH "Emergency Medical Services+" OR (emergenc* OR (acute N3 (care OR medicine)))) AND (MH child+ OR MH infant+ OR MH adolescence+ OR MH pediatrics+ OR MH Child Health Services+ OR MH Hospitals, Pediatric+ OR (adolescen* OR infan* OR newborn* OR (new N1 born*) OR baby OR babies OR neonat* OR child* OR kid OR kids OR toddler* OR teen* OR boy* OR girl* OR minors OR underag* OR (under N1 (age* OR aging)) OR juvenil* OR youth* OR kindergar* OR puber* OR pubescen* OR prepubescen* OR prepubert* OR pediatric* OR paediatric* OR school* OR preschool* OR highschool*)) AND (MH Drug Utilization+ OR MH Prescriptions, Drug+ OR MH Inappropriate Prescribing+ OR (((drug* OR antibiotic*) N3 ("use" OR usage OR registrat* OR consum* OR utiliz* OR pattern* OR reduc* OR decrease*)) OR prescri* OR overprescri* OR underprescri*))

**PubMed publisher**

((antibiotic*[tiab] OR antiinfect*[tiab] OR anti biotic*[tiab] OR anti infect*[tiab] OR ceftriaxone*[tiab] OR amoxicillin*[tiab] OR penicillin*[tiab] OR vancomycin*[tiab] OR cotrimoxazole*[tiab] OR ampicillin*[tiab] OR gentamicin*[tiab] OR clindamycin*[tiab] OR erythromycin*[tiab] OR cefotaxime*[tiab] OR ciprofloxacin*[tiab] OR dexamethasone*[tiab] OR prednisone*[tiab] OR azithromycin*[tiab] OR cytarabine*[tiab] OR cyclophosphamide*[tiab] OR macrolide*[tiab] OR doxycycline*[tiab])) AND ((fever OR febril*[tiab] OR hypertherm*[tiab])) AND ((emergenc*[tiab] OR (acute AND (care OR medicine)))) AND ((adolescen*[tiab] OR infan*[tiab] OR newborn*[tiab] OR (new born*[tiab]) OR baby OR babies OR neonat*[tiab] OR child*[tiab] OR kid OR kids OR toddler*[tiab] OR teen*[tiab] OR boy*[tiab] OR girl*[tiab] OR minors OR underag*[tiab] OR under ag*[tiab] OR juvenil*[tiab] OR youth*[tiab] OR kindergar*[tiab] OR puber*[tiab] OR pubescen*[tiab] OR prepubescen*[tiab] OR prepubert*[tiab] OR pediatric*[tiab] OR paediatric*[tiab] OR school*[tiab] OR preschool*[tiab] OR highschool*[tiab])) AND ((((drug*[tiab] OR antibiotic*[tiab]) AND ("use" OR usage OR registrat*[tiab] OR consum*[tiab] OR utiliz*[tiab] OR pattern*[tiab] OR reduc*[tiab] OR decrease*[tiab])) OR prescri*[tiab] OR overprescri*[tiab] OR underprescri*[tiab])) AND publisher[sb]

**Google scholar**

Antibiotic fever|hyperthermia|febrile emergency child|children|newborn|neonatal|adolescents|adolescence|pediatrics|pediatric|paediatrics|paediatric|childhood "drug|antibiotic use|usage|registration|consumption|pattern"|prescription|overprescription

Supplementary material 2. Quality assessment of individual studies

| **Study** | **MINORS score** |  | **Antibiotic prescription as primary outcome** |  | **N children reported antibiotics** |  | **Quality** |
| --- | --- | --- | --- | --- | --- | --- | --- |
| Angoulvant 2011 France | 11/14 | B | Yes | A | 53055 | A | High |
| Aronson 2015 USA | 12/16 | B | Yes | A | 1617 | A | High |
| Coco 2009 USA | 11/14 | B | Yes | A | 8325 | A | High |
| Craig 2010 Australia | 16/16 | A | No | B | 15781 | A | High |
| Irwin 2017 UK | 14/16 | B | Yes | A | 1101 | A | High |
| Jain 2014 USA | 18/24 | B | Yes | A | 19075 | A | High |
| Kornblith 2017 US | 11/14 | B | Yes | A | 2918 | A | High |
| Kronman 2010 USA | 11/14 | B | Yes | A | 266000 | A | High |
| Lacroix 2014 France | 24/24 | A | Yes | A | 271 | B | High |
| Linder 2005 USA | 11/14 | B | Yes | A | 4158 | A | High |
| Manzano 2009 Canada | 22/22 | A | Yes | A | 384 | B | High |
| Nelson 2016 US | 17/22 | B | Yes | A | 1610 | A | High |
| Ochoa 2001 Spain | 12/14 | B | Yes | A | 6249 | A | High |
| Ouldali 2017 | 19/22 | B | Yes | A | 196062 | A | High |
| Spiro 2004 USA | 19/22 | B | Yes | A | 681 | A | High |
| Spiro 2006 USA | 24/24 | A | Yes | A | 283 | B | High |
| Benin 2003 USA | 11/14 | B | Yes | A | 391 | B | Moderate |
| Bonner 2003 USA | 21/24 | B | Yes | A | 391 | B | Moderate |
| Brauner 2009 Israel | 18/24 | B | No | B | 148 | B | Moderate |
| Bustinduy 2017 UK | 12/14 | B | No | B | 1097 | A | Moderate |
| Chao 2007 USA | 22/24 | B | Yes | A | 206 | B | Moderate |
| Doan 2009 Canada | 22/24 | B | Yes | A | 200 | B | Moderate |
| Galetto-Lacour 2001 Switzerland | 14/16 | B | No | B | 124 | B | Moderate |
| Houten 2017 Netherlands | 14/16 | B | No | B | 577 | A | Moderate |
| Iyer 2006 USA21 | 23/24 | B | No | B | 700 | A | Moderate |
| Khine 2014 USA | 12/14 | B | Yes | A | 461 | B | Moderate |
| King 2007 USA | 11/14 | B | Yes | A | 442 | B | Moderate |
| Li-Kim-Moy 2016 Australia | 18/22 | B | Yes | A | 301 | B | Moderate |
| Massin 2006 Belgium | 13/16 | B | Yes | A | 376 | B | Moderate |
| McCormick 2005 USA | 23/24 | B | No | B | 209 | B | Moderate |
| Ong 2007 USA | 12/16 | B | Yes | A | 272 | B | Moderate |
| Planas 2011 Spain | 12/14 | B | No | B | 381 | B | Moderate |
| Ploin 2007 France | 13/16 | B | No | B | 538 | A | Moderate |
| Poehling 2006 USA | 20/22 | B | No | B | 468 | B | Moderate |
| Shah 2016 US | 11/14 | B | No | B | 1664 | A | Moderate |
| Trautner 2006 USA | 11/14 | B | No | B | 103 | B | Moderate |
| Vos-Kerkhof 2015 the Netherlands | 23/24 | B | No | B | 439 | B | Moderate |
| Wheeler 2001 USA | 18/24 | B | Yes | A | 144 | B | Moderate |
| Ahmed 2010 USA | 10/14 | C | Yes | A | 321 | B | Low |
| Ayanruoh 2009 USA | 17/24 | C | Yes | A | 8280 | A | Low |
| Benito-Fernández 2006 Spain | 16/22 | C | Yes | A | 206 | B | Low |
| Blaschke 2013 USA | 16/22 | C | Yes | A | 1166 | A | Low |
| Colvin 2012 USA | 17/22 | B | No | B | 75 | C | Low |
| Copp 2010 USA | 10/14 | C | Yes | A | 1828 | A | Low |
| Fischer 2009 USA | 11/16 | C | Yes | A | 144 | B | Low |
| Galetto-Lacour 2003 Switzerland | 13/16 | B | No | B | 99 | C | Low |
| Goldman 2008 Canada | 11/16 | C | No | B | 257 | B | Low |
| Isaacman 2001 USA | 10/14 | C | No | B | 577 | A | Low |
| Kilic 2012 Turkey | 9/14 | C | Yes | A | 2544 | A | Low |
| McCaig 2006 USA | 10/14 | C | No | B | 2100 | A | Low |
| Murray 2017 US | 16/22 | C | No | B | 520 | A | Low |
| Nibhanipudi 2016 US | 15/22 | C | No | B | 100 | B | Low |
| Özkaya 2009 Turkey | 18/22 | B | Yes | A | 97 | C | Low |
| Sharma 2002 USA | 18/22 | B | No | B | 72 | C | Low |
| Waddle 2008 USA | 16/22 | C | No | B | 423 | B | Low |
